# Epidemiology of fungal infection in COVID 19 in Spain during 2020 and 2021: a nationwide study

**DOI:** 10.1038/s41598-024-54340-1

**Published:** 2024-03-03

**Authors:** R. López-Herrero, L. Sánchez-de Prada, A. Tamayo-Velasco, M. Heredia-Rodríguez, M. Bardají Carrillo, P. Jorge Monjas, O. de la Varga-Martínez, S. Resino, G. Sarmentero-López de Quintana, E. Gómez-Sánchez, E. Tamayo

**Affiliations:** 1 BioCritic, Group for Biomedical Research in Critical Care Medicine, 47005 Valladolid, Spain; 2 Anesthesiology and Critical Care Department, Hospital Clínico Universitario de Valladolid, 47003 Valladolid, Spain; 3 Department of Surgery, Faculty of Medicine, Universidad de Valladolid, 47005 Valladolid, Spain; 4 Microbiology Department, Hospital Universitario Río Hortega, 47012 Valladolid, Spain; 5 Haematology and Hemotherapy Department, Hospital Clínico Universitario de Valladolid, 47003 Valladolid, Spain; 6 Centro de Investigación Biomédica en Red de Enfermedades Infecciosas (CIBERINFEC), Instituto de Salud Carlos III, Madrid, Spain; 7 Anesthesiology and Critical Care Department, Complejo Asistencial Universitario de Salamanca, 37007 Salamanca, Spain; 8 Department of Anesthesiology, Hospital Universitario Infanta Leonor, 28031 Madrid, Spain; 9 Unidad de Infección Viral e Inmunidad, Centro Nacional de Microbiología, Instituto de Salud Carlos III, Madrid, Spain

**Keywords:** COVID-19, Fungal infection, Spain, Hospitalization, Microbiology, Health care, Risk factors

## Abstract

We realize a nationwide population-based retrospective study to analyze the characteristics and risk factors of fungal co-infections in COVID-19 hospitalized patients as well as describe their causative agents in the Spanish population in 2020 and 2021. Data were obtained from records in the Minimum Basic Data Set of the National Surveillance System for Hospital Data in Spain, provided by the Ministry of Health, and annually published with two years lag. The assessment of the risk associated with the development of healthcare-associated fungal co-infections was assessed using an adjusted logistic regression model. The incidence of fungal co-infection in COVID-19 hospitalized patients was 1.41%. The main risk factors associated were surgery, sepsis, age, male gender, obesity, and COPD. Co-infection was associated with worse outcomes including higher in-hospital and in ICU mortality, and higher length of stay. *Candida spp.* and *Aspergillus spp.* were the microorganisms more frequent. This is the first study analyzing fungal coinfection at a national level in hospitalized patients with COVID-19 in Spanish population and one of the few studies available that demonstrate that surgery was an independent risk factor of Aspergillosis coinfection in COVID-19 patients.

## Introduction

The infection produced by SARS-COV2 represents a public health burden. To date, the global pandemic has caused 636,440,663 confirmed cases, including 6,606,624 deaths^1^. Although coinfections are not as common as those in other viral respiratory pneumonias, COVID-19 have been also described to suffer bacterial coinfections^2^.

Fungal coinfection is a well-known cause of complication in respiratory viral infections., but the incidence shows huge variations. For example, the incidence of Aspergillus-influenza virus coinfection ranges from 7 and 32%^3–5^. Before COVID-19, fungal infections associated with influenza pneumonia have been described to cause an increase in mortality and severity, with invasive pulmonary aspergillosis being the most common agent^6^. Additionally, previous coronavirus outbreaks of severe acute respiratory syndrome (SARS), patients with fungal coinfections also have higher mortality in both general (14.80–27%) and critically ill patients (21.90–33%)^7^. Therefore, the delay in diagnosis and treatment could be a determinant for survival^4^. Until now, the information known about fungal infections in COVID-19 patients is limited. In a prospective study carried out by Garcia-Vidal in Spain including 989 patients, fungal coinfection was observed in 0.70% of cases^8^. A meta-analysis found an incidence of 4% in fungal superinfections in COVID-19 patients^9^. Fungal superinfections are more frequent in COVID-19 patients admitted to the ICU, especially in those who are under mechanical ventilation^2^. Critical ill patients have increased proinflammatory and anti-inflammatory cytokines, as well as a decreased number of CD4 + and CD8 + lymphocyte counts^10,11^, that increase the risk of invasive fungal infection. The most common fungal coinfections in COVID-19 patients are pulmonary aspergillosis, invasive candidiasis, and mucormycosis^8,12–14^.

Different risk factors have been associated with fungal infections in patients with COVID-19, such as admission to the ICU, length of stay, co-morbidities like diabetes mellitus and use of high doses of steroids^15^. Early appropriate treatment in patients with risk factors is of importance to reduce the unnecessary use of antifungals, therefore reducing antifungal resistance and minimizing costs. Given that there are not many studies on fungal coinfections in patients with COVID-19, and the variability of the results, we propose to carry out a nationwide study that groups all COVID-19 patients based on the National Surveillance System for Hospital Data. The aim of the study was to describe the epidemiology and risk factors of fungal co-infections in COVID-19 hospitalized patients.

## Methods

### Study design and data source

A nationwide population-based retrospective study was performed in patients with diagnosis confirmed of COVID-19 during the first two years of the pandemic, 2020 and 2021 in Spain. The data was obtained from the records of the Minimum Basic Data Set (MBDS) of the National System of Hospital Data Surveillance in Spain, provided by the Ministry of Health, Consumer Affairs, and Social Welfare, published annually, published with a two-year lag. The MDBS is a clinical and administrative database, with an estimated coverage of 99.5% of hospital discharges registered in both public and private hospitals in Spain^16,17^. It comprises 20 diagnoses, each one indicating if the diagnosis was present on admission, and 20 therapeutic procedures according to the International Classification of Diseases 10th Revision, Clinical Modification (ICD-10-CM)^18^. The MDBS provides encrypted patient identification, sex, age, dates of hospital admission and discharge, information on intensive care unit (ICU) admission, length of stay in the ICU, diagnoses, and procedures during hospitalization, as well as the outcome at discharge. The data were handled with utmost confidentiality in strict compliance with Spanish legislation. It is important to point that when working with CMBD data, not all the clinical data that would be necessary are available. This study was approved by the CEIm Area de Salud Valladolid Este Ethics Review Board under the code PI 22–2855, which waived the informed consent requirement given the anonymous character of the data used in the study. All methods were performed in accordance with the relevant guidelines and regulations.

### Study variables

We selected all hospitalized patients in Spanish public and private hospitals between January 1, 2020, and December 31, 2021, with a confirmed primary diagnosis of COVID-19 (ICD-10-CM codes B97.29 and U07.1^19^. Additionally, we included all hospitalized patients with ICD-10-CM codes for fungal infections (Supplementary Table 1), acute organ dysfunction (Supplementary Table 2), and site of infection (Supplementary Table 3). The codes were adapted from Shen et al.^20^, Angus et al.^21^, and Dombovskiy et al.^22^.

The patients in our study were divided into two groups based on the presence of fungal coinfection and the absence of fungal coinfection (FI and NFI group, respectively). We studied the impact of fungal coinfections in hospitalized patients with COVID-19, as well as hospital and ICU mortality. In addition to collecting clinical information from patients admitted during this period, we constructed a logistic regression model to identify risk factors associated with developing a fungal infection.

### Model development and statistical analysis

The results were reported as median (interquartile range) for continuous variables and as percentages and frequencies for categorical variables. To compare continuous variables, the t-test or Mann–Whitney U test was used, and for comparing categorical variables, the chi-square test or Fisher's exact test was used, as required. Analyses were conducted to identify variables correlated with fungal infection.

Subsequently, a multivariate logistic regression analysis was performed using the backward stepwise Wald method, including variables that showed a *p* value < 0.1 in the previous analysis. Two-tailed tests were conducted, and Odds Ratios (OR) were reported with a 95% confidence interval and corresponding *p* values. A *p* value ≤ 0.05 was considered statistically significant. Due to the large number of potential risk factors, collinearity between explanatory variables was evaluated.

The statistical analysis for both studies was performed using Python 3.9 and SPSS Statistics version 27.0 (IBM Corp, Armonk, New York). All tests conducted were two-tailed, and *p* values < 0.05 were considered statistically significant.

## Results

### Patient cohorts

**Figure 1:**
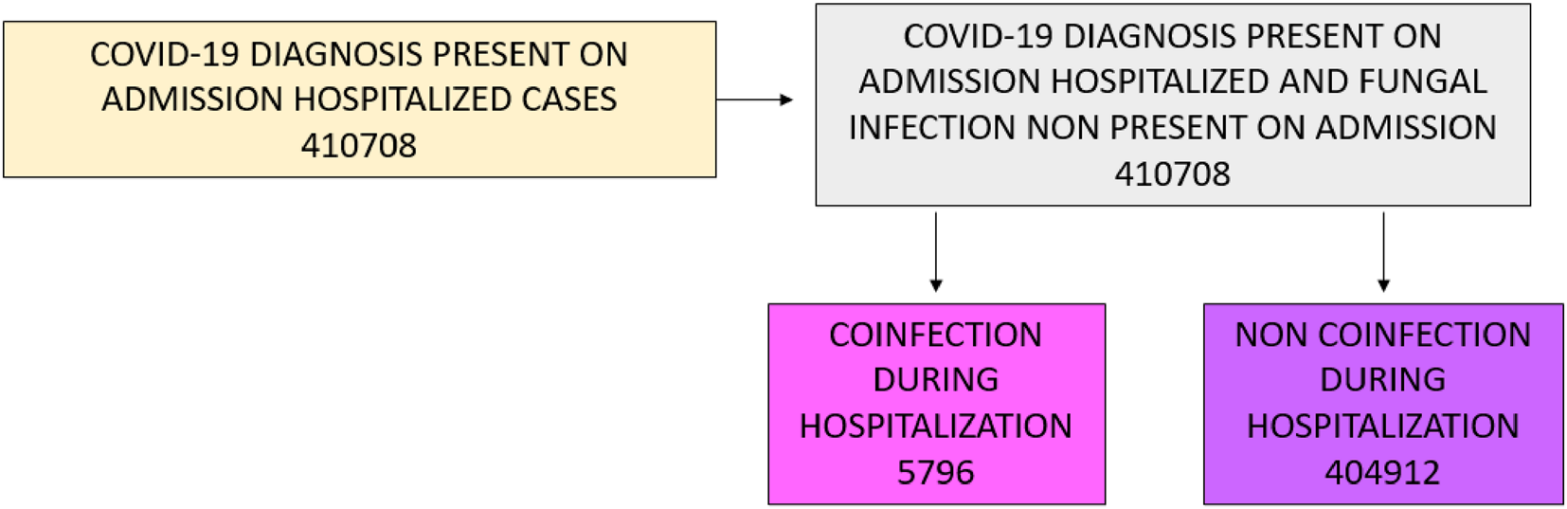
Study flowchart of patients with primary diagnosis of COVID-19 admitted in Spain from January 1, 2020, to December 31, 2021.

We identified a total of 410,708 patients with a primary diagnosis of COVID-19 and fungal infection non present on admission upon admission to Spanish public and/or private hospitals from January 1, 2020, to December 31, 2021. Among these, 5796 presented fungal infections during their hospitalization (1.41%) (Fig. 1).

Fungal infection was more frequently present in men (62.84% in FI group vs. 57.19% in NFI group), patients admitted to the ICU (68% in FI group vs. 10.13% in NFI group), patients with higher hospital mortality (34.54% in FI group vs. 14.44% in NFI group), and ICU mortality (41.11% in FI group vs. 26.77% in NFI group), as well as a higher need for invasive ventilatory support (67.41% in FI group vs. 6.61% in NFI group) and non-invasive ventilatory support (26.14% in FI group vs. 7.46% in NFI group) compared to those without concurrent fungal infection. Additionally, patients admitted to the hospital with fungal infection had a longer higher length of stay (LoS) (43.94 days in FI group vs. 10.59 days in NFI group), as well as in the ICU (36.05 days in FI vs. 15.86 days in NFI group), compared to those without fungal infection. Regarding comorbidities, those with fungal infection more frequently had diabetes mellitus (20.53% in FI group vs. 19.61% in NFI group), obesity (16.13% in FI group vs. 11.16% in NFI group), Chronic obstructive pulmonary disease (CPOD) (15.91% in FI group vs. 13.84% in NFI group), and cardiovascular diseases (27.35% in FI group vs. 25.13% in NFI group). Furthermore, patients in the FI group more frequently developed sepsis (62.47% in FI group vs. 7.81% in NFI group), with dysfunction of one organ (36.18% in FI group vs. 3.79% in NFI group), two organs (17.18% in FI group vs. 1.69% in NFI group), and more than two organs (7% in FI group vs. 0.61% in NFI group). The number of organs with failure was higher in the FI group (1.4 in FI group vs. 0.72 in NFI group). The organ most frequently affected by failure in the FI group was the respiratory system (89.61% in FI group vs. 45.35%), followed by the hematological system (13.34% FI group vs. 5.3% in NFI group) (Table 1).

**Table 1:** Characteristics of patients admitted in Spain during the years 2020 and 2021 due to SARS-CoV-2 infection, comparing fungal infection (FI) and non-fungal infection (NFI) groups.

	NFI	FI	*p* value
No.	404,912	5796	
Mean age (years)	65.39 (65.34; 65.45)	67.01 (66.69; 67.34)	< 0.001
Gender (male)	231,550 (57.19%)	3642 (62.84%)	< 0.001
Charlson index	1.31 (1.31; 1.32)	1.54 (1.49; 1.59)	< 0.001
No comoborbidities	199,657 (49.31)	2247 (38.77)	< 0.001
> 2 comoborbidities	76,825 (18.97)	1309 (22.58)	< 0.001
2 comoborbidities	68,517 (16.92)	1231 (21.24)	< 0.001
1 comoborbidities	59,913 (14.8)	1009 (17.41)	< 0.001
Morbidities			
Arterial hypertension	165,096 (40.77%)	2336 (40.30%)	0.478
Cardiovascular disease	101,767 (25.13%)	1585 (27.35%)	< 0.001
Diabetes	79,410 (19.61%)	1190 (20.53%)	< 0.001
Obesity	45,172 (11.16%)	935 (16.13%)	< 0.001
Chronic obstructive pulmonary disease	56,055 (13.84%)	922 (15.91%)	< 0.001
Renal disease	27,641 (6.83%)	374 (6.45%)	0.274
Liver disease	21,236 (5.24%)	321 (5.54%)	0.334
Cancer	17,556 (4.34%)	275 (4.74%)	0.001
Abuse of tobacco	15,028 (3.70%)	153 (2.64%)	< 0.001
Peripheral vascular disease	2380 (0.59%)	31 (0.53%)	0.662
Digestive system diseases	1152 (0.28%)	24 (0.41%)	0.067
Human immunodeficiency virus	858 (0.21%)	14 (0.24%)	0.731
Organ Failure			
Number of organ failure	0.72 (0.71; 0.72)	1.4 (1.38; 1.42)	< 0.001
1 organ	171,947 (42.47)	3371 (58.16)	< 0.001
2 organs	44,555 (11.0)	1489 (25.69)	< 0.001
> 2 organs	9061 (2.24)	543 (9.37)	< 0.001
No organ failure	179,349 (44.29)	393 (6.78)	< 0.001
Organ Failure			
Respiratory	183,636 (45.35%)	5194 (89.61%)	< 0.001
Hematologic	21,473 (5.3%)	773 (13.34%)	< 0.001
Renal	43,648 (10.78%)	587 (10.13%)	0.117
Metabolic	6836 (1.69%)	480 (8.28%)	< 0.001
Hepatic	14,623 (3.61%)	387 (6.68%)	< 0.001
Cardiovascular	5407 (1.34%)	363 (6.26%)	< 0.001
Neurologic	13,908 (3.43%)	306 (5.28%)	< 0.001
Surgery	9352 (2.31%)	2370 (40.89%)	< 0.001
Sepsis	31,605 (7.81%)	3621 (62.47%)	< 0.001
Sepsis + 1 organ failure	15,362 (3.79%)	2097 (36.18%)	< 0.001
Sepsis + 2 organ failure	6863 (1.69%)	996 (17.18%)	< 0.001
Sepsis + > 2 organ failure	2473 (0.61%)	406 (7.0%)	< 0.001
Invasive ventilatory support	26,782 (6.61%)	3907 (67.41%)	< 0.001
Non-invasive ventilatory support	30,196 (7.46%)	1515 (26.14%)	< 0.001
ICU admission	40,999 (10.13%)	3941 (68.0%)	< 0.001
ICU length of stay	15.86 (15.68; 16.05)	36.05 (35.15; 36.95)	< 0.001
Length of stay (days)	10.59 (10.55; 10.63)	43.94 (43.06; 44.81)	< 0.001
ICU death	10,976 (26.77%)	1620 (41.11%)	< 0.001
In-hospital death	58,467 (14.44%)	2002 (34.54%)	< 0.001

Meaning of the acronyms: ICU: intensive care unit. Values are expressed as absolute number (percentage) and mean (standard deviation). A patient can be reflected in several categories of the same variable.

The respiratory tract was the most frequent site of infection in both patient groups (97.43% in FI vs. 86.34% in NFI). In the FI group, genitourinary infections were also predominant (40.1%) (Table 2). Regarding the profile of fungal infections, candidiasis was the most frequent coinfection, with mucocutaneous candidiasis being the most common (44.65%), followed by invasive candidiasis (18.65%). Coinfections caused by Aspergillus spp. also stood out, with an incidence of 21.91% (Table 2).

**Table 2:** Site of infection and fungus family in patients with SARS-CoV-2 infection, comparing comparing fungal infection (FI) and non-fungal infection (NFI) groups.

	NFI	FI	*p* value
Site infection			
Respiratory	349,611 (86.34%)	5647 (97.43%)	< 0.001
Genitourinary	19,944 (4.93%)	2324 (40.1%)	< 0.001
Digestive	2216 (0.55%)	218 (3.76%)	< 0.001
Circulatory	470 (0.12%)	23 (0.40%)	< 0.001
Nervous	68 (0.02%)	5 (0.09%)	0.001
Fungus family			
Mucocutaneous candidiasis	0 (0.00)	2588 (44.65%)	< 0.001
Aspergillosis	0 (0.00)	1270 (21.91%)	< 0.001
Invasive candidiasis	0 (0.00)	1081 (18.65%)	< 0.001
Unspecified candidiasis	0 (0.00)	1038 (17.91%)	< 0.001
Candidiasis of the skin	0 (0.00)	259 (4.47%)	< 0.001
Unspecified mycosis	0 (0.00)	90 (1.55%)	< 0.001
Dermatophytosis	0 (0.00)	19 (0.33%)	< 0.001
Zygomycosis	0 (0.00)	5 (0.09%)	< 0.001
Cryptococcosis	0 (0.00)	1 (0.02%)	< 0.001
Resistance to antifungals	0 (0.00)	4 (0.07%)	< 0.001

Values are expressed as absolute number (percentage) and mean (standard deviation).

### Risk factors of fungal coinfection

**Figure 2:**
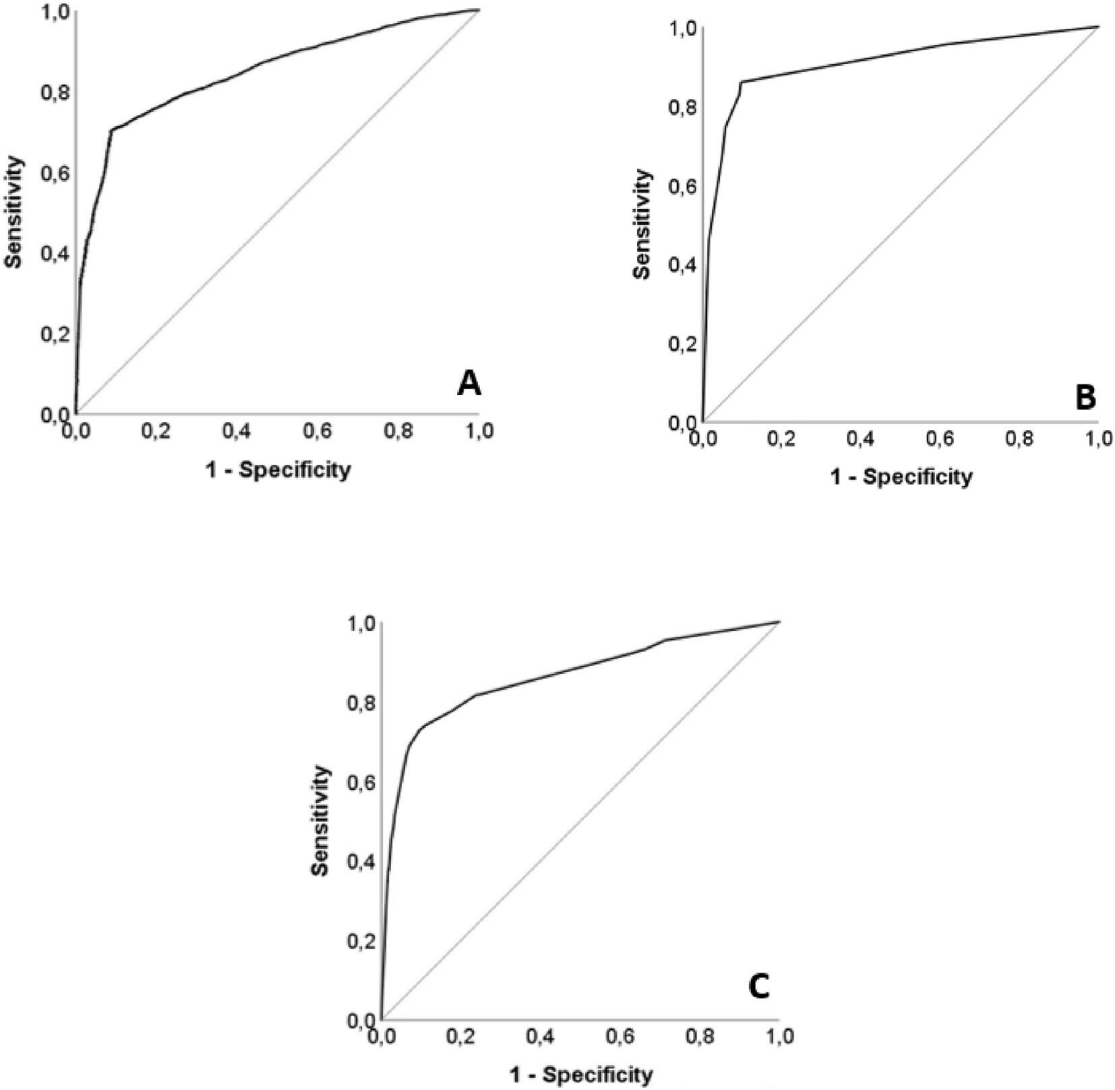
(**A**) The area under the curve of the fungal infection group was 0.844 (95% CI 0.838–0.850). (**B**) The AUC of the invasive candidiasis infection was 0.907 (95% CI 0.895–0.918). (**C**) The AUC of the invasive aspergillus infection was 0.860 (95% CI 0.847–0.872).

Sepsis increases the likelihood of developing a fungal infection by 10.094 times (adjusted OR 10.094, 95% CI 9.478–10.748) (Supplementary Table 4). Additionally, undergoing surgery increases the odds of fungal infection by 8.793 times (adjusted OR 8.793, 95% CI 8.220–9.406). Male gender has a 1.004 times higher probability of fungal infection than females (adjusted OR 1.004, 95% CI 1.002–1.006). The presence of COPD raises the risk by 1.286 times (adjusted OR 1.286, 95% CI 1.191–1.388). Lastly, being obese increases the risk of fungal infection by 1.338 times (adjusted OR 1.338, 95% CI 1.237–1.446) (Supplementary Table 4). The ROC curve of fungal infection group (receiver operating characteristic curve) has an area under the curve (AUC) of 0.844 (95% CI 0.838–0.850) (Fig. 2A).

### Infections caused by invasive candidiasis: epidemiology and risk factors

We conducted an analysis of the characteristics of patients who presented with invase candidiasis (CI group) and compared them with patients who did not have candidiasis (NCI group) (Supplementary table 5).

The presence of sepsis increases the probability of developing invasive candidiasis by 13.882 times (adjusted OR 13.882, 95% CI 11.818–16.306). Additionally, undergoing surgery increases the odds by 10.288 times (adjusted OR 10.288, 95% CI 8.914–11.874), and being male increases it by 1.506 times (adjusted OR 1.506, 95% CI 1.312–1.727) (Supplementary Table 6). The ROC curve of fungal infection group (receiver operating characteristic curve) has an area under the curve (AUC) of 0.907 (95% CI 0.895–0.918) (Fig. 2B).

### Infections caused by invasive aspergillosis: epidemiology and risk factors

Subsequently, we conducted an analysis of the characteristics of patients who presented with Aspergillosis (AI group) and compared them with patients who did not have Aspergillosis (NAI group) (Supplementary table 7).

Surgery increases the probability of developing Aspergillus infection by 10.658 times (adjusted OR 10.658, 95% CI 9.285 to 12.235), while the presence of sepsis increases the odds of developing the infection by 6.476 times (adjusted OR 6.476, 95% CI 5.642–7.432). Male sex has a 1.648 times higher probability of fungal infection than females (adjusted OR 1.648, 95% CI 1.451 to 1.871). The presence of COPD raises the risk by 1.466 times (adjusted OR 1.466, 95% CI 1.265 to 1.698), and obesity shows an increase of 1.428 times (adjusted OR 1.428, 95% CI 1.230 to 1.659). Lastly, having cancer increases the risk by 1.299 times (adjusted OR 1.299, 95% CI 1.018 to 1.657). (Supplementary Table 8). The ROC curve of fungal infection group (receiver operating characteristic curve) has an area under the curve (AUC) of 0.860 (95% CI 0.847–0.872) (Fig. 2C).

## Discussion

This is a retrospective study of COVID-19 hospitalized patients in Spain during 2020 and 2021 with a nosocomial fungal coinfection. To the best of our knowledge, this is the first study analyzing fungal coinfection at a national level in hospitalized patients with COVID-19 in Spanish population. We found that (i) The incidence of fungal coinfections in patients with COVID-19 was 1.41%, (ii) Patients with fungal coinfection had a longer general hospital stay, higher ICU admission, mortality and stay, as well as and a greater need for mechanical ventilation, (iii) The respiratory tract was the most frequent site of infection, followed by the genitourinary tract, (iv) Risk factors for the development of fungal infection included surgery, sepsis, age, male sex, obesity and chronic obstructive pulmonary disease.

Previous studies analyzing the association between fungal coinfections and SARS-CoV-2, reported highly variable incidences, ranging from 0.70% in a study by García-Vidal^8^ to 26.70% in another study by White^23^. In our study, the incidence found was 1.41%, which is within the lower range of the previously described incidences. Estimating the coinfection rate in all COVID-19 cases is a challenging task as not all patients undergo sequential testing for coinfection. Also, this disparity could be attributed to the different methodologies used in these studies (case series, retrospective studies, prospective studies) with varying numbers of patients, and conducted in different populations.

Critically ill patients have a higher risk of developing coinfections. These patients exhibit increased levels of pro-inflammatory and anti-inflammatory markers, elevated cytokine levels, and reduced CD4 + and CD8 + lymphocyte levels^10,24^. These conditions, along with invasive procedures performed in the ICU^25^, and prolonged hospital stays^26^, increased the risk of developing fungal coinfections. In our study, patients with fungal coinfection had longer hospital stay, higher risk of death, increased ICU admission, and longer duration of ICU stay.

Furthermore, the most commonly fungal microorganisms found in the population studied, were Candida and Aspergillus that have been previously described^2,27^. In our cohort, the most frequent site of infection was the respiratory tract. This could be explained by the destruction of the respiratory tract epithelium and suppression of the immune response in the site after viral infections. Also, the antibiotic treatment and the microbiota alteration, may lead to the emergence of fungal infection^13^. The second most frequent site of infection was the genitourinary tract. This could be attributed to the presence of risk factors in COVID-19 patients that promote the occurrence of fungal infections in the urinary tract, such as renal failure, advanced age, diabetes, cancer, and immune system disorders^28^.

Multiple factors have been associated with the risk of fungal coinfection in COVID-19 patients, such as admission to the ICU, high-dose steroid administration, presence of diabetes mellitus, COPD, among other^15,29,30^. In our study, the main risk factors described were surgery, sepsis, obesity, advanced age, men gender, and COPD.

Surgery, especially abdominal surgery, causes damage to the body's natural barrier, which can favor gastrointestinal translocation and lead to Candida infection, as Candida often colonizes the intestines^31^. This risk factor has not been described in previous studies conducted in COVID-19 patient groups, but in the literature, different risk assessment scales analyze the main risk factors for developing invasive fungal infection in non-COVID-19 patients, such as the scale developed by León et al.^32^, where surgery was identified as a significant risk factor, consistent with our study findings. This is the first time that surgery has been described as a risk factor for the development of aspergillosis in patients with SARS-CoV-2. The presence of surgery as a risk factor for the development of aspergillosis in patients with SARS-CoV-2 may not have been previously identified due to the lack of information on surgical procedures in previous studies or because this variable was not considered in those studies. Therefore, further research and data collection are required to better understand the relationship between surgery and fungal infections in COVID-19 patients.

Sepsis and the presence of multiorgan dysfunction further contribute to the occurrence of superinfections due to the immunosuppressive state they induce. Patients with sepsis and multiorgan failure have multiple risk factors for developing fungal infections, such as antibiotic administration, invasive therapeutic strategies^33^, sepsis-induced immunosuppression^34^, and intestinal barrier dysfunction^35^. The presence of sepsis was a significant risk factor for *Aspergillus spp.* infection in COVID-19 patients, which is consistent with the findings of the meta-analysis by Chong et al.^36^.

Obese patients have an increased risk of bacterial, viral, and fungal infections^37,38^ due to the inflammatory state they experience, which leads to elevated proinflammatory cytokines and favors the development of chronic inflammation. Additionally, obese patients often require invasive mechanical ventilation, which further contributes to the development of superinfections^39^.

We observed that age was independently associated with an increased incidence of fungal infection. Advanced age is a known risk factor for infections due to immunosenescence, which results in reduced activation of the immune system (increased immature T lymphocytes, altered CD4 + /CD8 + T cell ratio, and decreased immune response), thus promoting the occurrence of infections^40,41^.

We report that fungal coinfection was more frequent in men than in women. This higher risk in males could be influenced by hormonal factors, genetic factors leading to differences in immune response, or even genetic polymorphisms.

In this research, the presence of COPD was identified as a risk factor for the development of *Aspergillus spp.* infection in COVID-19 patients, that do not present in patients with invasive candidiasis. This could be explained by the fact that *Aspergillus* spp. spores are normally cleared by the ciliary action of the respiratory epithelium, but in COPD patients (as in those infected with SARS-CoV-2), this ciliary clearance is impaired, facilitating the invasion of the bronchial mucosa and lung parenchyma^42^. Additionally, COPD patients often receive inhaled corticosteroid treatment, which can reduce immune activity and predispose them to fungal infection.

Our study presents certain limitations due to its retrospective design, relying on data obtained from the Spanish MBDS. As with any retrospective analysis, there is a possibility of under-coding of variables, leading to incomplete or inaccurate information. This could introduce potential bias and affect the robustness of our findings. Furthermore, the lack of coding for certain analytical variables and multiple admissions of the same patient might have influenced the precision and completeness of our results. Despite the limitations, our study possesses notable strengths. Foremost among these is the substantial sample size, which confers high statistical power and enhances the reliability of our analyses. The extensive dataset enabled us to provide a comprehensive and representative perspective on the epidemiological landscape of fungal co-infections in patients with COVID-19 within the Spanish population. This large-scale approach contributes to a more nuanced understanding of the prevalence and characteristics of fungal infections in this specific context.

## Conclusion

In summary, this study reported the characteristics and risk factors of COVID-19 patients in the Spanish population during the years 2020 and 2021. Our results showed that the incidence was 1,41% and these patients presented higher in-hospital and length of stay in intensive care unit and mortality, as well as intensive care unit admission. Surgery, chronic obstructive pulmonary disease, sepsis, gender male, and advanced age were the main risk factors. Also, this is one of the few studies available that demonstrate that surgery was an independent risk factor of *Aspergillus spp*. coinfection in COVID-19 patients.

### Supplementary Information

Supplementary Tables.

## Data Availability

The MDBS is the property of the Ministry of Health. Therefore, any researcher can request the data related to this article from the Ministry of Health by email (icmbd@msssi.es), by fax (+ 34,915,964,111), or by mail (Instituto de Información Sanitaria, Área de información y Estadisticas Asistenciales, Ministerio de Sanidad, Consumo y Bienestar Social. Paseo del Prado 18–20; 28,071 Madrid. Spain).

## References

[1] COVID-19 cases | WHO COVID-19 dashboard. datadot https://data.who.int/dashboards/covid19/cases.

[2] Pemán J (2020). Fungal co-infection in COVID-19 patients: Should we be concerned?. Rev. Iberoam. Micol..

[3] Beumer MC (2019). Influenza virus and factors that are associated with ICU admission, pulmonary co-infections and ICU mortality. J. Crit. Care.

[4] Schauwvlieghe AFAD (2018). Invasive aspergillosis in patients admitted to the intensive care unit with severe influenza: A retrospective cohort study. Lancet Respir. Med..

[5] Schwartz IS (2020). High rates of influenza-associated invasive pulmonary aspergillosis may not be universal: A retrospective cohort study from Alberta, Canada. Clin. Infect. Dis. Off. Publ. Infect. Dis. Soc. Am..

[6] Salazar F, Bignell E, Brown GD, Cook PC, Warris A (2022). Pathogenesis of respiratory viral and fungal coinfections. Clin. Microbiol. Rev..

[7] Stockman LJ (2007). Severe acute respiratory syndrome in children. Pediatr. Infect. Dis. J..

[8] Garcia-Vidal C (2021). Incidence of co-infections and superinfections in hospitalized patients with COVID-19: A retrospective cohort study. Clin. Microbiol. Infect. Off. Publ. Eur. Soc. Clin. Microbiol. Infect. Dis..

[9] Peng J (2021). Fungal co-infection in COVID-19 patients: Evidence from a systematic review and meta-analysis. Aging.

[10] Tay MZ, Poh CM, Rénia L, MacAry PA, Ng LFP (2020). The trinity of COVID-19: Immunity, inflammation and intervention. Nat. Rev. Immunol..

[11] Chen N (2020). Epidemiological and clinical characteristics of 99 cases of 2019 novel coronavirus pneumonia in Wuhan, China: A descriptive study. Lancet Lond. Engl..

[12] Lansbury L, Lim B, Baskaran V, Lim WS (2020). Co-infections in people with COVID-19: A systematic review and meta-analysis. J. Infect..

[13] Guan W-J (2020). Clinical characteristics of coronavirus disease 2019 in China. N. Engl. J. Med..

[14] Koehler P (2020). COVID-19 associated pulmonary aspergillosis. Mycoses.

[15] Tiseo G (2022). Risk factors and outcomes of fungal superinfections in patients with severe COVID-19: An observational study from Pisa academic hospital. Infez. Med..

[16] Ministerio de Sanidad - Sanidad en datos - Registro de Altas de los Hospitales del Sistema Nacional de Salud. CMBD. https://www.sanidad.gob.es/estadEstudios/estadisticas/cmbdhome.htm.

[17] BOE-A-2015-1235 Real Decreto 69/2015, de 6 de febrero, por el que se regula el Registro de Actividad de Atención Sanitaria Especializada. https://www.boe.es/buscar/act.php?id=BOE-A-2015-1235.

[18] The Web’s Free 2024 ICD-10-CM/PCS Medical Coding Reference. https://www.icd10data.com/.

[19] Ministerio de Sanidad - Sanidad en datos - Ediciones anteriores de CIE-10-ES. https://www.sanidad.gob.es/estadEstudios/estadisticas/normalizacion/Ed_Anteriores_CIE_10_ES.htm.

[20] Shen H-N, Lu C-L, Yang H-H (2010). Epidemiologic trend of severe sepsis in Taiwan from 1997 through 2006. Chest.

[21] Angus DC (2001). Epidemiology of severe sepsis in the United States: Analysis of incidence, outcome, and associated costs of care. Crit. Care Med..

[22] Dombrovskiy VY, Martin AA, Sunderram J, Paz HL (2007). Rapid increase in hospitalization and mortality rates for severe sepsis in the United States: A trend analysis from 1993 to 2003. Crit. Care Med..

[23] White PL (2021). A national strategy to diagnose coronavirus disease 2019-associated invasive fungal disease in the intensive care unit. Clin. Infect. Dis. Off. Publ. Infect. Dis. Soc. Am..

[24] Chen G (2020). Clinical and immunological features of severe and moderate coronavirus disease 2019. J. Clin. Invest..

[25] Singh G, Pitoyo CW, Aditianingsih D, Rumende CM (2016). Risk factors for early invasive fungal disease in critically ill patients. Indian J. Crit. Care Med. Peer-Rev Off. Publ. Indian Soc. Crit. Care Med..

[26] Yang X (2020). Clinical course and outcomes of critically ill patients with SARS-CoV-2 pneumonia in Wuhan, China: A single-centered, retrospective, observational study. Lancet Respir. Med..

[27] Hoenigl M (2022). COVID-19-associated fungal infections. Nat. Microbiol..

[28] Wise GJ, Talluri GS, Marella VK (1999). Fungal infections of the genitourinary system: Manifestations, diagnosis, and treatment. Urol. Clin. North Am..

[29] Nucci M (2021). Increased incidence of candidemia in a tertiary care hospital with the COVID-19 pandemic. Mycoses.

[30] Du Y (2020). Clinical features of 85 fatal cases of COVID-19 from Wuhan. A retrospective observational study. Am. J. Respir. Crit. Care Med..

[31] Kumamoto CA (2011). Inflammation and gastrointestinal Candida colonization. Curr. Opin. Microbiol..

[32] León C (2006). A bedside scoring system ("Candida score’’) for early antifungal treatment in nonneutropenic critically ill patients with Candida colonization. Crit. Care Med..

[33] Thomas-Rüddel DO, Schlattmann P, Pletz M, Kurzai O, Bloos F (2022). Risk factors for invasive candida infection in critically ill patients: A systematic review and meta-analysis. Chest.

[34] Hotchkiss RS, Monneret G, Payen D (2013). Sepsis-induced immunosuppression: From cellular dysfunctions to immunotherapy. Nat. Rev. Immunol..

[35] Meng M, Klingensmith NJ, Coopersmith CM (2017). New insights into the gut as the driver of critical illness and organ failure. Curr. Opin. Crit. Care.

[36] Chong WH, Saha BK, Neu KP (2022). Comparing the clinical characteristics and outcomes of COVID-19-associate pulmonary aspergillosis (CAPA): A systematic review and meta-analysis. Infection.

[37] Frasca D (2016). Obesity decreases B cell responses in young and elderly individuals. Obes. Silver Spring Md.

[38] Petrova D (2020). La obesidad como factor de riesgo en personas con COVID-19: Posibles mecanismos e implicaciones. Aten. Primaria.

[39] Simonnet A (2020). High prevalence of obesity in severe acute respiratory syndrome coronavirus-2 (SARS-CoV-2) requiring invasive mechanical ventilation. Obes. Silver Spring Md.

[40] Castle SC (2000). Clinical relevance of age-related immune dysfunction. Clin. Infect. Dis. Off. Publ. Infect. Dis. Soc. Am..

[41] Yoshikawa TT (2000). Epidemiology and unique aspects of aging and infectious diseases. Clin. Infect. Dis. Off. Publ. Infect. Dis. Soc. Am..

[42] Bulpa P, Dive A, Sibille Y (2007). Invasive pulmonary aspergillosis in patients with chronic obstructive pulmonary disease. Eur. Respir. J..

