# Supplementary material for: Epidemiology of fungal infection in COVID 19 in Spain during 2020 and 2021: a nationwide study
Source: Sci Rep. 2024 Mar 3;14:5203. doi: 10.1038/s41598-024-54340-1 (PMC10909879; doi:10.1038/s41598-024-54340-1)
Supplement: Supplementary file 1 — Supplementary Tables. [file 41598_2024_54340_MOESM1_ESM.docx]

**Supplementary Tables**

| B37.5 | Candidal meningitis | Invasive candidiasis |
| --- | --- | --- |
| B37.6 | Candidal endocarditis | Invasive candidiasis |
| B37.7 | Candidal sepsis | Invasive candidiasis |
| B37.8 | Candidiasis of other sites | Unspecified candidiasis |
| B37.9 | Candidiasis, unspecified | Unspecified candidiasis |
| B44.0 | Invasive pulmonary aspergillosis | Aspergillosis |
| B44.1 | Other pulmonary aspergillosis | Aspergillosis |
| B44.2 | Tonsillar aspergillosis | Aspergillosis |
| B44.7 | Disseminated aspergillosis | Aspergillosis |
| B44.8 | Other forms of aspergillosis | Aspergillosis |
| B44.9 | Aspergillosis, unspecified | Aspergillosis |
| B45.0 | Pulmonary cryptococcosis | Cryptococcosis |
| B45.1 | Cerebral cryptococcosis | Cryptococcosis |
| B45.2 | Cutaneous cryptococcosis | Cryptococcosis |
| B45.3 | Osseous cryptococcosis | Cryptococcosis |
| B45.7 | Disseminated cryptococcosis | Cryptococcosis |
| B45.8 | Other forms of cryptococcosis | Cryptococcosis |
| B45.9 | Cryptococcosis, unspecified | Cryptococcosis |
| B46.0 | Pulmonary mucormycosis | Zygomycosis |
| B46.1 | Rhinocerebral mucormycosis | Zygomycosis |
| B46.2 | Gastrointestinal mucormycosis | Zygomycosis |
| B46.3 | Cutaneous mucormycosis | Zygomycosis |
| B46.4 | Disseminated mucormycosis | Zygomycosis |
| B46.5 | Mucormycosis, unspecified | Zygomycosis |
| B46.8 | Other zygomycoses | Zygomycosis |
| B46.9 | Zygomycosis, unspecified | Zygomycosis |
| B49 | Unspecified mycosis | Unspecified mycoses |
| Z16.32 | Resistance to antifunfal drug (s) |  |

**Supplementary Table 1:** International Classification of Diseases, 10th Revision, Clinical Modification (ICD-10-CM) codes f sepsis for fungal causal agents

| **Organ System** | **ICD-10-CM Code** | **ICD-10-CM Code Description** |
| --- | --- | --- |
| **Cardiovascular** | I46 | Cardiac arrest |
|  | I95.1 | Orthostatic hypotension |
|  | I95.89 | Other specified hypotension |
|  | I95.9 | Hypotension, unspecified |
|  | R57.0 | Shock without mention of trauma |
|  | R57.9 | Hypotension, transient |
| **Hematologic** | D65 | Disseminated intravascular coagulation (Defibrination syndrome) |
|  | D68 | Other and unspecified coagulation defects |
|  | D69 | Purpura and other bleeding conditions |
|  | R79.1 | Abnormal coagulation profile |
| **Hepatic** | K72.0 | Acute and subacute necrosis of liver |
|  | K72.01, K72.91 | Hepatic encephalopathy |
|  | K75.9 | Hepatitis (septic & not elsewhere classified) |
|  | K76.3 | Hepatic infarction |
| **Neurologic** | F06 | Transient organic psychosis |
|  | G93.1 | Anoxic brain damage |
|  | G93.4 | Other and unspecified types of encephalopathy |
|  | G31.2 | Alcoholic encephalopathy |
|  | G94 | Other disorders of the brain in diseases classified elsewhere |
|  | I67.4 | Hypertensive encephalopathy |
|  | R40 | Drowsiness, stupor and coma |
|  | 4A00 | Electroencephalography |
| **Renal** | N00 | Acute glomerulonephritis |
|  | N17 | Acute renal failure |
|  | N19 | Renal shutdown, renal failure unspecified |
|  | 5A1D | Hemodialysis |
| **Respiratory** | J95 | Intraoperative and postprocedural complications and disorders of the respiratory system, not elsewhere classified |
|  | R06.81 | Apnea |
|  | R09.2 | Respiratory arrest |
|  | J96 | Respiratory insufficiency |
|  | 5A19, 5A09 | Ventilator management |
|  | [0BH1](javascript:loadCT(%220BH1%22,%22cie10pcs%22,%222012%22)) | Endotracheal intubation (emergency procedure) |
|  | A09357, 5A09557, 5A09457 | Continuous positive airway pressure |
| **Metabolic** | E87.2 | Acidosis, metabolic or lactic |

**Supplementary Table 2**: International Classification of Diseases, 10th Revision, Clinical Modification (ICD-10-CM) codes for acute organ dysfunction

|  | **Nervous** |
| --- | --- |
| A17 | Tuberculosis of meninges and central nervous system |
| A39.0 | Meningococcal infection |
| A51.41 | Acute syphilitic meningitis (secondary) |
| A52.1 | symptomatic neurosyphilis |
| A52.2 | Asymptomatic neurosyphilis |
| A52.3 | Unspecified neurosyphilis |
| G00 | Bacterial meningitis, not elsewhere classified |
| G04.2 | Bacterial meningoencephalitis and meningomyelitis, not elsewhere classified |
| B45.1 | Cerebral cryptococcosis |
| G02 | Meningitis in other infectious and parasitic diseases classified elsewhere |
| G06 | Central nervous system abscess |
| G08 | Phlebitis of intracranial sinus |
| H44.0 | Purulent endophthalmitis |
| H05.00 | Acute inflammation of orbit |
| H60.20 | Malignant otitis externa |
| H70.0 | Acute mastoiditis |
|  | **Circulatory** |
| A52.00-A52.04 | Cardiovascular syphilis |
| A52.06 | Cardiovascular syphilis |
| A52.09 | Cardiovascular syphilis |
| A54.83 | gonococcal heart infection (endocarditis, pericarditis, myocarditis) |
| I01.2 | Acute rheumatic myocarditis |
| I30 | Acute pericarditis |
| I33 | Acute or subacute endocarditis |
|  | **Respiratory** |
| A15 | respiratory tuberculosis |
| A31.0 | Pulmonary diseases due to other mycobacteria |
| A36 | Diphtheria |
| A38 | Streptococcal throat/scarlet fever |
| A54.5 | Gonococcal infection of pharynx |
| B37.1 | Candidiasis, of lung |
| B38.0 | Acute pulmonary coccidioidomycosis |
| B38.1 | Chronic pulmonary coccidioidomycosis |
| B39.5 | Histoplasma duboisii pneumonia |
| B39.2 | Histoplasma capsulatum pneumonia |
| B39.9 | Histoplasmosis pneumonia unspecified |
| B45 | Cryptococcus neoformans |
| B44 | Aspergillosis |
| B59 | Pneumocystosis |
| J01 | Acute sinusitis |
| J02 | Acute pharyngitis |
| J03 | Acute tonsillitis |
| J04 | Acute laryngitis/tracheitis |
| J06 | Acute upper respiratory infection of multiple sites/not |
| J36 | Peritonsillar abscess |
| J12 | Viral pneumonia |
| J13 | Pneumococcal pneumonia |
| J15 | Other bacterial pneumonia |
| J16 | Pneumonia due to another specified organism |
| J18 | Pneumonia, organism not otherwise specified |
| J10 | Influenza |
| J44.1 | Acute exacerbation of obstructive chronic bronchitis |
| J47 | Bronchiectasis |
| J86 | Pyothorax |
| J85 | Abscess of lung and mediastinum |
|  | **Digestive** |
| A00 | Cholera |
| A01 | Typhoid/paratyphoid fever |
| A02 | Other salmonella infection |
| A03 | Shigellosis |
| A05 | Other food poisoning |
| A04.0-A04.4 | Intestinal infections due to *Escherichia coli* |
| A04.8 | Other specified bacterial intestinal infections |
| A04.9 | Bacterial intestinal infection, unspecified |
| A18.3 | Tuberculosis of intestines peritoneum and mesenteric glands |
| B82 | Intestinal parasitism unspecified |
| K04.7 | Periapical abscess without sinus |
| K04.6 | Periapical abscess with sinus |
| M27.2 | Inflammatory conditions of the jaw |
| K11.3 | Abscess of the salivary glands |
| K12.2 | Cellulitis and abscess of oral soft tissue |
| K35 | Acute appendicitis |
| K37 | Appendicitis not otherwise specified |
| K36 | Other appendicitis |
| K57.12 | Diverticulitis of the small intestine without haemorrhage |
| K57.13 | Diverticulitis of the small intestine with haemorrhage |
| K57.32 | Diverticulitis of colon without haemorrhage |
| K57.33 | Diverticulitis of colon with haemorrhage |
| K61.0 | Anal abscess |
| K61.1 | rectal abscess |
| K61.3 | Ischiorectal abscess |
| K65 | Peritonitis |
| K63.0 | Intestinal abscess |
| K94.02 | Infection of colostomy |
| K94.12 | Infection of enterostomy |
| K63.1 | Perforation of intestine |
| K75.0 | Abscess of liver |
| K75.1 | Portal pyaemia |
| K81.0 | Acute cholecystitis |
|  | **Genitourinary** |
| A18.1 | Tuberculosis of genitourinary system |
| A54.24 | Gonococcal salpingitis specified as acute |
| B37.4 | Candidiasis of other urogenital sites |
| N10 | Kidney infection |
| N39.0 | Urinary tract infection not otherwise specified |
| N41 | Prostatic inflammation |
| N45 | Orchitis and epididymitis |
| N73 | Female pelvic inflammation disease |
| N71 | Uterine inflammatory disease |
| N75.1 | Abscess of Bartholin’s gland |
| N76.4 | Other abscess of vulva |
|  | **Pregnancy** |
| O03 | Spontaneous abortion |
| O04 | Complications after (induced) termination of pregnancy |
| O08 | Complications following abortion and ectopic and molar pregnancies |
| O23 | Infections of genitourinary tract in pregnancy |
| O41.1 | Infection of amniotic cavity |
| O85 | Major puerperal infection |
| N61.1 | Abscess of breast |
|  | **Skin, soft tissue, or bone** |
| A02.24 | Salmonella osteomyelitis |
| A18.0 | Tuberculosis of bones and joints |
| A18 | Tuberculosis of other organs |
| A31.1 | Cutaneous diseases due to other mycobacteria |
| A146 | Erysipelas |
| A39.83 | Meningococcal arthropathy |
| A48.0 | Gas gangrene |
| A52.77 | Syphilis of bone |
| A54.5 | Gonococcal infection of joint |
| L03 | Cellulitis and acute lymphangitis |
| L05.01 | Pilonidal cyst, with abscess |
| L08 | Other local skin infection |
| M00 | Pyogenic arthritis |
| M72.6 | Necrotizing fasciitis |
| M86 | Osteomyelitis |
|  | **Other** |
| R78.81 | Bacteraemia |
| T79.8XXA | Post-traumatic wound infection, not elsewhere classified |
| T82.7 | Infection or inflammation of device/graft |
| T81. 4 | Postoperative infection |

**Supplementary Table 3**: International Classification of Diseases, 10th Revision, Clinical Modification (ICD-10-CM) codes for the site of infection causing sepsis”.

|  | **aOR (CI95%)** | **p-value** |
| --- | --- | --- |
| **Sepsis** | 10.094 (9.478-10.748) | <0.001 |
| **Surgery** | 8.793 (8.220-9.406) | <0.001 |
| **Obesity** | 1.338 (1.237-1.446) | <0.001 |
| **Chronic obstructive pulmonary disease** | 1.286 (1.191-1.388) | <0.001 |
| **Gender male** | 1.105 (1.042-1.171) | <0.001 |
| **Age** | 1.004 (1.002-1.006) | <0.001 |

**Supplementary Table 4:** Independent risk factors for the onset of fungal infection. Stepwise logistic regression model. Meaning of the acronyms: aOR: adjusted odds ratio, CI 95%: 95% confidence interval.

|  | **NCI** | **CI** | **p-value** |
| --- | --- | --- | --- |
| **Gender (male)** | 234414 (57,23%) | 778 (71,97%) | <0.001 |
| **Mean Age (years)** | 65.42 ± 18,17 | 64.87 ± 10,39 | 0.084 |
| **Charlson Index**  0 comorbidities  2 comorbidities  ≥3comorbidities  1 comorbidity | 213107 (52.02%)  68109 (16.63%)  68939 (16.83%)  59472 (14.52%) | 624 (57.72%)  201 (18.59%)  129 (11.93%)  127 (11.75%) | <0.001 |
| **Comorbidities** |  |  |  |
| Arterial hypertension | 166040 (40.53%) | 397 (36.73%) | 0.011 |
| Diabetes | 80403 (19.63%) | 197 (18.22%) | 0.246 |
| Obesity | 45943 (11.22%) | 164 (15.17%) | <0.001 |
| Chronic obstructive pulmonary disease | 56837 (13.88%) | 140 (12.95%) | 0.380 |
| Heart disease | 29584 (7.22%) | 56 (5.18%) | 0.010 |
| Liver disease | 17222 (4.20%) | 38 (3.52%) | 0.259 |
| Cancer | 17794 (4.34%) | 37 (3.42%) | 0.138 |
| Abuse of tobacco | 15148 (3.70%) | 33 (3.10%) | 0.261 |
| Peripheral vascular disease | 9210 (2.25%) | 17 (1.57%) | 0.134 |
| Digestive system diseases | 1174 (0.29%) | 2 (0.19%) | 0.776 |
| Human immunodeficiency virus | 864 (0.21%) | 2 (0.19%) | 1.000 |
| **Organ failure** |  |  |  |
| 1 organ failure | 174509 (42.60%) | 634 (58.65%) | <0.001 |
| 2 organ failure | 45334 (11.07%) | 296 (27.38%) | <0.001 |
| ≥3 organ failure | 9340 (2.28%) | 139 (12.86%) | <0.001 |
| No organ failure | 180444 (44.05%) | 12 (1.11%) | <0.001 |
| **Mechanical ventilation** | 29722 (7.26%) | 967 (89.45%) | <0.001 |
| **Sepsis** | 34411 (8.40%) | 815 (75.39%) | <0.001 |
| **Surgery** | 11112 (2.71%) | 610 (56.43%) | <0.001 |
| **Length of stay (days)** | 10.95 ± 12.82 | 52.40 ± 36.35 | <0.001 |
| **In-hospital death** | 59970 (14.64%) | 499 (46.16%) | <0.001 |

**Supplementary Table 5:** Characteristics of patients admitted in Spain during the years 2020 and 2021 due to SARS-CoV-2 infection, comparing candida infection (CI) and non-candida infection (NFI) groups. Values are expressed as absolute number (percentage) and mean (standard deviation).

|  | **aOR (CI95%)** | **p-value** |
| --- | --- | --- |
| **Sepsis** | 13.882 (11.818-16.306) | <0.001 |
| **Surgery** | 10.288 (8.914-11.874) | <0.001 |
| **Gender Male** | 1.506 (1.312-1.727) | <0.001 |

**Supplementary Table 6:** Independent risk factors for the onset of invasive candidiasis infection. Stepwise logistic regression model. Meaning of the acronyms: aOR: adjusted odds ratio, CI 95%: 95% confidence interval.

|  | **NAI** | **AI** | **p-value** |
| --- | --- | --- | --- |
| **Gender (male)** | 234266 (57.22%) | 926 (72.91%) | <0.001 |
| **Mean Age (years)** | 65.42 ± 18.17 | 65.40 ± 10.18 | 0.951 |
| **Charlson Index**  0 comorbidities  2 comorbidities  1 comorbidity  ≥3comorbidities | 213118 (52.05%)  68056 (16.62%)  59382 (14.50%)  68882 (16.82%) | 613 (48.27%)  254 (20%)  217 (17.09%)  186 (14.65%) | <0.001 |
| **Comorbidities** |  |  |  |
| Arterial hypertension | 165962 (40.53%) | 475 (37.40%) | 0.023 |
| Diabetes | 80355 (19.63%) | 245 (19.29%) | 0.765 |
| Chronic obstructive pulmonary disease | 56749 (13.86%) | 228 (17.95%) | <0.001 |
| Obesity | 45885 (11.21%) | 222 (17.48%) | <0.001 |
| Heart disease | 29566 (7.22%) | 74 (5.83%) | 0.055 |
| Cancer | 17759 (4.34%) | 72 (5.67%) | 0.020 |
| Liver disease | 17194 (4.20%) | 66 (5.20%) | 0.077 |
| Abuse of tobacco | 15137 (3.70%) | 44 (3.50%) | 0.661 |
| Peripheral vascular disease | 9190 (2.24%) | 37 (2.91%) | 0.108 |
| Human immunodeficiency virus | 860 (0.21%) | 6 (0.47%) | 0.054 |
| Digestive system diseases | 1171 (0.29%) | 5 (0.39%) | 0.422 |
| **Organ failure** |  |  |  |
| 1 organ failure | 174402 (42.60%) | 741 (58.35%) | <0.001 |
| 2 organs failure | 45276 (11.06%) | 354 (27.87%) | <0.001 |
| ≥3 organs failure | 9325 (2.28%) | 154 (12.13%) | <0.001 |
| No organ failure | 180435 (44.07%) | 21 (1.65%) | <0.001 |
| **Mechanical ventilation** | 29590 (7.23%) | 1099 (86.54%) | <0.001 |
| **Sepsis** | 34469 (8.42%) | 757 (59.61%) | <0.001 |
| **Surgery** | 11112 (2.71%) | 610 (48.03%) | <0.001 |
| **Length of stay (days)** | 10.95 ± 12.86 | 46.65 ± 31.85 | <0.001 |
| **In-hospital death** | 59779 (14.6%) | 690 (54.33%) | <0.001 |

**Supplementary Table 7:** Characteristics of patients admitted in Spain during the years 2020 and 2021 due to SARS-CoV-2 infection, comparing Aspergillus infection (AI) and non-Aspergillus infection (NAI) groups. Values are expressed as absolute number (percentage) and mean (standard deviation).

|  | **aOR (CI95%)** | **p-value** |
| --- | --- | --- |
| **Surgery** | 10.658 (9.285-12.235) | <0.001 |
| **Sepsis** | 6.476 (5.642-7.432) | <0.001 |
| **Gender Male** | 1.648 (1.451-1.871) | <0.001 |
| Chronic obstructive pulmonary disease | 1.466 (1.265-1.698) | <0.001 |
| **Obesity** | 1.428 (1.230-1.659) | <0.001 |
| **Cancer** | 1.299 (1.018-1.657) | 0.035 |

**Supplementary Table 8:** Independent risk factors for the onset of invasive aspergillosis infection. Stepwise logistic regression model. Meaning of the acronyms: aOR: adjusted odds ratio, CI 95%: 95% confidence interval.
